# Editorial: Controlling psychometric measures for method effects by means of factor analysis

**DOI:** 10.3389/fpsyg.2022.984050

**Published:** 2022-07-18

**Authors:** Christine DiStefano, Karl Schweizer, Stefan Troche

**Affiliations:** ^1^Department of Educational Studies, University of South Carolina, Columbia, MO, United States; ^2^Institute of Psychology, Goethe University Frankfurt, Frankfurt, Germany; ^3^Department of Psychology, University of Bern, Bern, Switzerland

**Keywords:** method effect, contextual effect, factor analysis, validity, measurement

As promoted by the most recent edition of the *Testing Standards for Psychological and Educational Testing* (American Educational Research Association et al., 2014), a unified approach to validity encourages test developers and test users to consider evidence in support of a construct along multiple domains. While evidence in support of score inferences is evaluated, there are many threats to validity that simultaneously exist. The presence of construct irrelevant variance is one such threat. This is defined as the introduction of extraneous, uncontrolled variables which cloud a researcher's view of the focal construct (Bandalos, 2018). When construct irrelevant variance is present, the meaningfulness and accuracy of the results can be adversely affected, which in turn can impact decisions made from the data and also may reduce the validity associated with score inferences.

In many educational and psychological testing situations, the introduction of method effects may be one of common occurrences of construct irrelevant variance. Method effects occur when variance associated with the method used to measure a construct is incorporated into the testing situation. These effects may arise from many sources, such as respondent acquiescence, social desirability, or inclusion of both positively and negatively worded items on the same form to name but a few. Other situations that arise in method variance have been defined and studied in the measurement literature.

Various psychometric techniques have been used isolate method effect variance (e.g., mixture modeling, multi-level modeling, item response theory, etc.); however, the focus of this Research Topic is on the use of factor analysis as the primary tool to control and account for method effects. Factor analysis allows for partitioning of the substantive component from the method effects component by modeling variance associated with the method effect in a manner such that it is distinct of the content under study (Marsh, 1989; Marsh and Grayson, 1995), thus allowing a clearer view of the main construct.

The Research Topic, “*Controlling Psychometric Measures for Method Effects by Means of Factor Analysis*” provides an opportunity for researchers to showcase current work in this area. The articles selected for this Research Topic examine the presence of method effects under a wide range of conditions. These include: a variety of data types, such as simulated data, empirical data, and data collected *via* “online” platforms (even in situation where automatized “bots” may provide data); different analysis frameworks, such as principal components analysis and bifactor models; and a variety of content areas outside of method effects (e.g., behavioral inhibition/behavioral activation, emotional intelligence, and motivation-development). On the face, it may look like the set of articles does not have much in common. Yet, the works highlight a multi-faceted view of method effects and illustrate to readers a few of the many ways in which method effects may be observed in practice. These varied situations remind readers that validity impairment due to a method effect is an all-time present danger. In addition, the Research Topic of articles shows the utility of the factor analytic framework, and provide a sample of different types of models which may be used to control the influence of method effects, as well as suggestions concerning fit criteria useful for evaluation, reflections of design conditions (e.g., varying sample sizes, number of items exhibiting method effects) on parameter bias and other evaluation criteria. While the focus is primarily on the use of factor analysis as the psychometric tool, the Research Topic of articles reminds researchers that construct irrelevant variance poses a threat to our scientific investigations, the resulting interpretations which may arise from confounded data and the advancement of science.

## Author contributions

All authors listed have made a substantial, direct, and intellectual contribution to the work and approved it for publication.

## Conflict of interest

The authors declare that the research was conducted in the absence of any commercial or financial relationships that could be construed as a potential conflict of interest.

## Publisher's note

All claims expressed in this article are solely those of the authors and do not necessarily represent those of their affiliated organizations, or those of the publisher, the editors and the reviewers. Any product that may be evaluated in this article, or claim that may be made by its manufacturer, is not guaranteed or endorsed by the publisher.
